# Real-time measurement of cellular bioenergetics in fully differentiated human nasal epithelial cells grown at air-liquid-interface

**DOI:** 10.1152/ajplung.00414.2019

**Published:** 2020-04-08

**Authors:** Emily Mavin, Bernard Verdon, Sean Carrie, Vinciane Saint-Criq, Jason Powell, Christian A. Kuttruff, Chris Ward, James P. Garnett, Satomi Miwa

**Affiliations:** ^1^Institute of Cellular Medicine, Newcastle University, Newcastle Upon Tyne, United Kingdom; ^2^Institute for Cell and Molecular Biosciences, Newcastle University, Newcastle Upon Tyne, United Kingdom; ^3^Institute of Health and Society, Newcastle University, Newcastle Upon Tyne, United Kingdom; ^4^Medicinal Chemistry, Boehringer Ingelheim Pharma, Biberach an der Riss, Germany; ^5^Immunology and Respiratory Diseases Research, Boehringer Ingelheim Pharma, Biberach an der Riss, Germany

**Keywords:** airway epithelium, glucose, metabolism, Seahorse

## Abstract

Shifts in cellular metabolic phenotypes have the potential to cause disease-driving processes in respiratory disease. The respiratory epithelium is particularly susceptible to metabolic shifts in disease, but our understanding of these processes is limited by the incompatibility of the technology required to measure metabolism in real-time with the cell culture platforms used to generate differentiated respiratory epithelial cell types. Thus, to date, our understanding of respiratory epithelial metabolism has been restricted to that of basal epithelial cells in submerged culture, or via indirect end point metabolomics readouts in lung tissue. Here we present a novel methodology using the widely available Seahorse Analyzer platform to monitor real-time changes in the cellular metabolism of fully differentiated primary human airway epithelial cells grown at air-liquid interface (ALI). We show increased glycolytic, but not mitochondrial, ATP production rates in response to physiologically relevant increases in glucose availability. We also show that pharmacological inhibition of lactate dehydrogenase is able to reduce glucose-induced shifts toward aerobic glycolysis. This method is timely given the recent advances in our understanding of new respiratory epithelial subtypes that can only be observed in vitro through culture at ALI and will open new avenues to measure real-time metabolic changes in healthy and diseased respiratory epithelium, and in turn the potential for the development of novel therapeutics targeting metabolic-driven disease phenotypes.

## INTRODUCTION

Metabolic phenotypes are influenced by a number of genetic and environmental factors including gender, age, diet, microbiome, exercise, hormones and medication. Each unique combination, and therefore unique metabolic phenotype, can determine the disease risk of an individual. This is of particular importance in respiratory disease research; recent studies have demonstrated that altered metabolic phenotypes can modulate disease-driving cellular processes prevalent in the pathologies of chronic lung diseases, such as cellular proliferation, differentiation, apoptosis, autophagy, senescence and inflammation (12, 28, 29). Despite the high metabolic activity of the lung, with glucose metabolism in particular surpassing that of many other organs (12), our understanding of metabolic dysfunction as a driver or consequence of respiratory disease pathology is limited.

Much of the metabolic activity of the lung occurs in the epithelium where cells have dense apical concentrations of mitochondria and carry out energy demanding processes such as mucin/surfactant production and mucociliary clearance (27). The respiratory epithelium provides a barrier to prevent nutrients entering the respiratory tract from the interstitium/blood and through tight control of glucose transport the glucose concentration in the airway is maintained at 3- to 20-fold lower than in plasma (6). In healthy airway epithelium, glucose is rapidly processed by hexokinase-dependent and -independent metabolic pathways to maintain low intracellular glucose concentrations (1). However, airway glucose concentrations are elevated in respiratory disease and hyperglycemia, which is associated with increased risk of respiratory infection (2, 18, 19). Thus, understanding the metabolic processes underlying lung metabolite homeostasis in health and disease could identify new therapeutic targets for the treatment of lung infections. The Seahorse platform is an excellent tool for interrogating cellular metabolism, measuring oxygen consumption rate (OCR) and extracellular acidification rate (ECAR), in real time. Recent studies modeling the human airway epithelium have utilized this technology to show how mitochondrial dysfunction, caused by cigarettes and e-cigarettes, might contribute to the pathology of lung disease (3, 23) as well as investigating how infection influences epithelial cell metabolism (13). While these studies all provide key information on airway cell metabolism, the data is all generated using epithelial cells grown in submerged cultures. Accordingly, a methodological development to monitor real time metabolic changes of fully differentiated airway epithelial cells, under a range of conditions, would be of particular benefit to the field of respiratory research.

Since Whitcutt et al. (25) first described a method to grow airway epithelial cells at an air-liquid interface (ALI) this has become widely used to investigate epithelial barrier function. ALI cultures are an excellent research resource as once the cells fully differentiate they have a range of features which are not present in undifferentiated cells (25). They form polarized barriers with characteristic epithelial ion transport properties and contain a heterogeneous cell population of secretory and ciliated cells which only become apparent in vitro when the cells are maintained at ALI. Recent single cell profiling has further demonstrated the heterogeneity of ALI cultures at a molecular level and allowed the authors to identify a rare and novel epithelial cell type, the pulmonary ionocyte (20). This highlights the importance of studying a heterogeneous mixture of differentiated cells, which makes our exploration of epithelial metabolism in ALI cultures particularly timely.

There are very few studies which have attempted to measure cellular metabolism in differentiated airway epithelial cells, and the only report which we are aware required the in-house manufacture of cell culture equipment (27), which is beyond the resources of many researchers. We describe a novel method utilizing the Seahorse XF24 Analyzer platform to monitor real time changes in the cellular bioenergetics of human airway epithelial cells fully differentiated at an ALI. We describe a simple protocol, predicated on inexpensive and widely available equipment.

## METHODS

### 

#### Sample source.

We obtained approval for the collection of clinical waste material obtained during routine nasal surgical procedures [Newcastle Biobank application NB-169, Research Ethics Committee Reference 17/NE/0361]. Written informed consent was obtained from all participants.

#### Epithelial cell culture at air-liquid interface.

Primary human airway epithelial cells were isolated from clinical waste material obtained during routine nasal surgical procedures. Chopped sections of epithelial tissue (~1 mm^3^) were cultured in PneumaCult-Ex Plus Medium (STEMCELL Technologies) for 7–9 days in Type I collagen-coated flasks (Purecol 30 µg/ml). P1 cells were then transferred onto collagen-coated 0.4-μm pore size transwells (Costar) at 150,000 cells/cm^2^. Once the cells were fully confluent (2–4 days) apical media was removed and basolateral media was switched to PneumaCult-ALI version 2 custom medium (STEMCELL Technologies) and the cells were maintained at ALI until fully differentiated (25). Barrier integrity of ALI cultures was monitored by measuring trans-epithelial electrical resistance (EVOM 2, World Precision Instruments) and ion channel function assessed using Ussing chamber short circuit current measurements. Transwells were routinely fixed with 2% glutaraldehyde for transmission electron microscopy (TEM)/scanning electron microscope (SEM) (Newcastle University Electron Microscopy Research Services) and 4% PFA for H&E staining (NHS Cellular Pathology, RVI Newcastle).

#### Seahorse XF24 analyzer.

All reagents were obtained from Sigma, unless stated otherwise. Apical washes, with prewarmed PBS, of the ALI cultures were carried out 24 h before all seahorse experiments. Sections of ALI cultures on Transwells were cut from the membrane using a 3-mm punch biopsy (Kai Medical). These were carefully loaded into a seahorse islet capture plate (Agilent Technologies) and held in place with the islet capture grid (see Fig. 2). Wells were loaded with 450-μl experimental media (DMEM without NaHCO_3_, glucose or phenol red (Sigma), supplemented with 1 mM d-glucose and 2 mM l-glutamine, pH 7.4 at 37°C). The plate was incubated at 37°C without CO_2_ while the cartridge was loaded and calibrated in XF24 Seahorse Analyzer (Agilent).

Cellular energetics for ATP production rates by mitochondrial oxidative phosphorylation and glycolysis were calculated by using the OCR and ECAR (16). Unbuffered medium enables the Seahorse Analyzer to accurately detect changes in acidification rates of the medium, as lactate secretion associated with glycolysis causes an acidification of the medium. Changes in OCR, after sequential inhibition of different stages of the electron transport chain, are used to measure mitochondrial ATP production (16). Oligomycin inhibits ATP synthase, thus oligomycin-induced changes in OCR reflect the oxygen consumption due to mitochondrial ATP production, which is used to calculate the mitochondrial ATP production rates. Carbonyl cyanide-4-(trifluoromethoxy)phenylhydrazone (FCCP) is an uncoupler, hence stimulating electron transport chain and increasing OCR. Rotenone and antimycin A, complex I and complex III inhibitors respectively, block electron transport activity and hence mitochondrial OCR and the OCR in the presence of them indicates nonmitochondrial respiration.

Injection ports for the cartridge were prepared in experimental buffer as follows. Injection port A with 50 μl of 1 mM, 14 mM, or 140 mM d-glucose (final concentrations to be 1, 5, or 15 mM). Injection port B with 55 μl of 50 μg/ml oligomycin (final concentration 5 μg/ml as determined by titration shown in Fig. 3). Injection port C with 60 μl of 25 μM FCCP (final concentration 2.5 μM, as determined by titration in Fig. 3) and injection port D with 65 μl of 25 μM antimycin A and 5 μM Rotenone (final concentrations 2.5 μM and 0.5 μM, respectively). Lactate dehydrogenase 5 (LDH5) inhibitor [synthesized according to the procedures described in patent WO 2015/140133 A1 (22)] was injected to a final concentration of 30 µM per well using DMSO vehicle control where appropriate.

Cycles of mix (2 min), wait (1 min), measure (3 min) were used. After our optimization experiments, we allowed 5 cycles for the cells to equilibrate, 7 cycles after glucose injection, 11 cycles after oligomycin, 7 cycles after FCCP, and 6 cycles after antimycin A and Rotenone addition. For LDH5 inhibition assays, we allowed 5 cycles for equilibration, 7 cycles after glucose injection (port A), 13 cycles each after LHD5inh injection (port B) and oligomycin (port C) with a final 7 cycles after antimycin A and Rotenone (port D) injection.

Data analysis to calculate absolute ATP production rates was carried out using the methods described by Mookerjee and Brand (16), taking into account the acidification rates due to mitochondrial CO_2_ production. All statistical analysis was carried out using GraphPad Prism 8.

## RESULTS

### 

#### Culture of ALI epithelial cells.

**Fig. 1.**
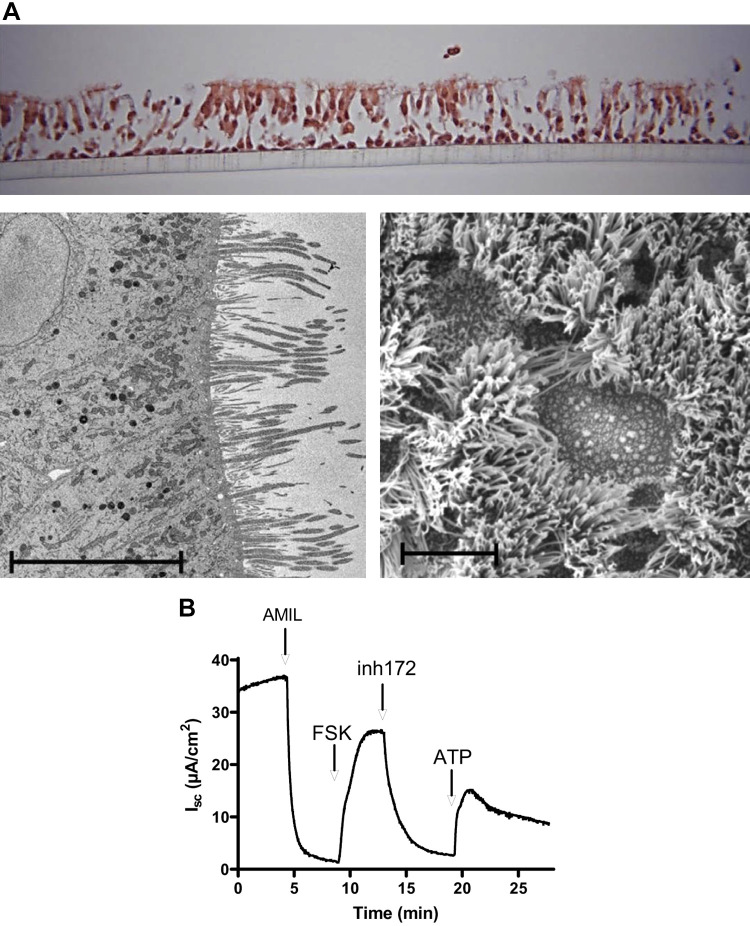
Nasal epithelial air-liquid interface (ALI) culture characterization. *A*: hematoxylin and eosin stain, transmission electron microscopy (TEM), and scanning electron microscope (SEM) images of apical side of ALI culture. Scale bars indicate 10 µm. *B*: short circuit current trace from Ussing chamber experiment of ALI culture showing conventional airway epithelial responses to amiloride (AMIL), forskolin (FSK), CFTR inhibitor-172 (inh172) (all at 10 µM) and ATP (100 µM). Chemical modulators were applied sequentially to the apical compartment of the Ussing chamber. *I*_sc_, short circuit current.

Primary human nasal epithelial cells were successfully grown at ALI to form fully differentiated pseudostratified cultures (Fig. 1*A*). Cilia were visible on the apical side (Fig. 1*A*) and cells were shown to contain a large number of apical mitochondria (Fig. 1*A*). Our ALI cultures showed electrophysiological polarization and had characteristic ion transport systems as measured in an Ussing system (Fig. 1*B*). The initial short circuit current (*I*_sc_) was completely inhibited by amiloride (10 µM) indicating that this standing *I*_sc_ is carried by apical to basolateral Na^+^ transport through apical epithelial sodium channels (ENaC). Further addition of forskolin (10 µM) generated a positive *I*_sc_ which reached a plateau and was fully reversed with CFTRinh-172, indicating apical Cl^−^ secretion via CFTR channels. Finally, addition of ATP (100 µM) generated a further positive *I*_sc_ (which decayed spontaneously). This is likely to be Cl^−^ efflux through apical Ca^2+^-activated Cl^−^ channels, activated by intracellular Ca^2+^ pulse (initiated via ATP interaction with apical P2Y receptors).

#### Seahorse analyzer protocol optimisation.

**Fig. 2.**
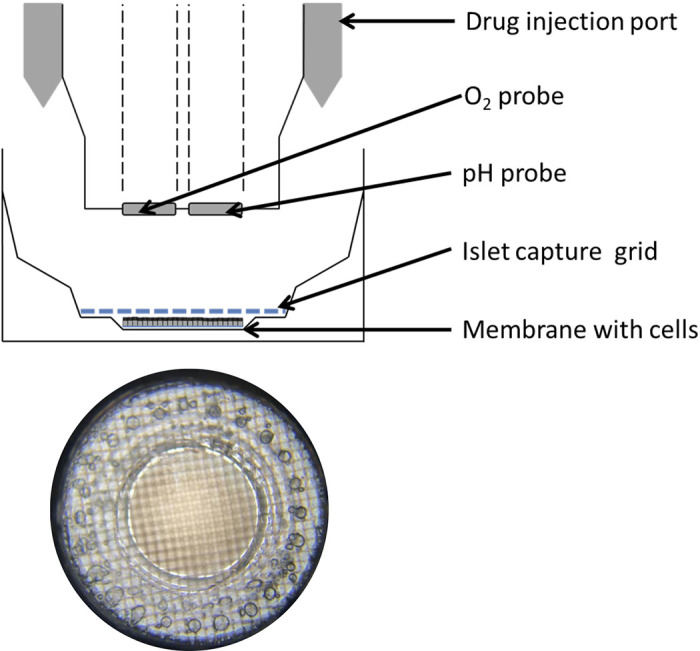
Schematic representation of air-liquid interface (ALI) culture in Seahorse islet capture plate.

**Fig. 3.**
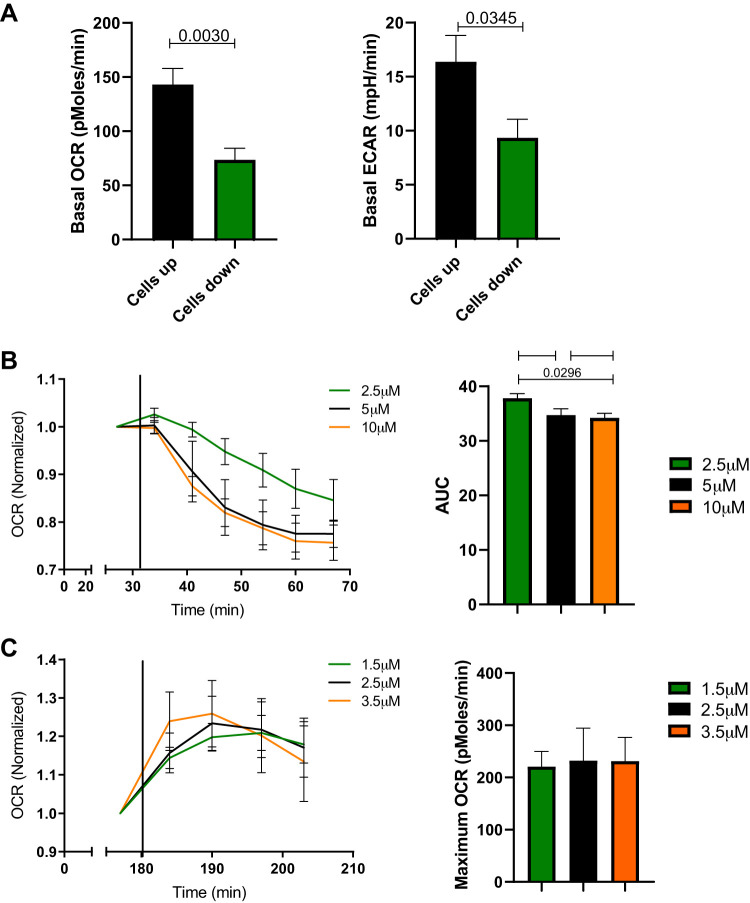
Protocol optimization for Seahorse XF24 Analyzer. *A*: comparison of air-liquid interface (ALI) cultures loaded into Islet capture plate with cells facing up or down in 10 mM d-glucose. Bar graphs indicate basal levels of oxygen consumption rate (OCR) and extracellular acidification rate (ECAR), before injection of any drugs. Error bars indicate means ± SE for 7–9 replicates and *P* values shown calculated by unpaired *t* test. *B*: oligomycin titration, OCR measurements normalized to last time point before addition of oligomycin (*left*), area-under-the-curve analysis of OCR (*right*), error bars indicate means ± SE for 5–7 replicates and *P* values calculated by one-way ANOVA. *C*: carbonyl cyanide-4-(trifluoromethoxy)phenylhydrazone (FCCP) titration, OCR normalized to last time point before addition of FCCP (*left*), maximum OCR in each well after addition of FCCP (*right*), error bars indicate means ± SE for 5–6 replicates and *P* values calculated by one-way ANOVA.

Sections of membrane with cells attached were loaded into the seahorse islet plate and held in place with the islet capture grid (Fig. 2). We compared the oxygen consumption rate (OCR) and extracellular acidification rate (ECAR) values when cells were loaded into the islet plate with the cells either facing up or down. Significantly higher values for both OCR and ECAR were obtained when cells were facing up (Fig. 3*A*). Therefore, it was decided that for all subsequent experiments disks of membrane would be loaded into the plate with the cells facing up. Epithelial cells remained attached to the membrane throughout the experiment as verified by visual inspection with brightfield microscopy.

Oligomycin concentrations ranging from 2.5 to 10 µM were tested. Each concentration was sufficient to induce a decrease in OCR as mitochondrial ATP synthesis was inhibited. The maximum change in OCR was seen in 5 µg/ml oligomycin without further decrease at 10 µg/ml (Fig. 3*B*), therefore 5 µg/ml was selected for all subsequent experiments. The FCCP concentration to induce maximum OCR was also determined (1.5–3.5 µM) and it was found 2.5 µM FCCP was sufficient to achieve the highest increase in OCR (Fig. 3*C*). Importantly, we found that it takes much longer for the pseudostratified cultures to achieve steady state after glucose and drug injections than a simple monolayer of airway epithelial cells (7).

#### ALI cultures at altered glucose concentrations.

**Fig. 4.**
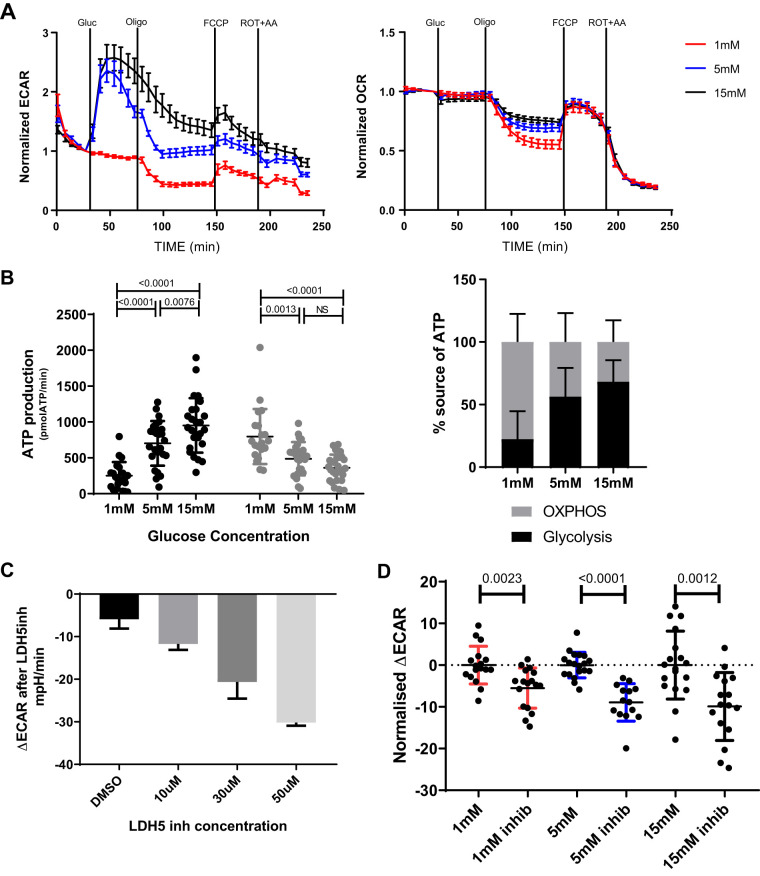
Metabolic profiling of air-liquid interface (ALI) cells at altered glucose concentrations. *A*: extracellular acidification rate (ECAR) (*left*) and oxygen consumption rate (OCR) (*right*) normalized to last reading before addition of glucose. Data from 4 to 6 repeats at each glucose concentration for 4 donors. Error bars indicate means ± SE. Gluc, glucose; FCCP, carbonyl cyanide-4-(trifluoromethoxy)phenylhydrazone; Rot, Rotenone; AA, antimycin A. *B*: absolute ATP production rates (*left*) and ATP production as a percentage of source, *P* values calculated by 2-way ANOVA (4–6 repeats at each glucose concentration from 6 donors). *C*: dose-dependent change in maximum ECAR before and after addition of LDH5 inhibitor, all at 15 mM d-glucose (3–5 repeats at each glucose concentration from 2 donors). *D*: change in maximum ECAR after addition of 30 µM lactate dehydrogenase 5 (LDH5) inhibitor at 1, 5, and 15 mM d-glucose, normalized to the average of control wells without inhibitor, *P* values calculated by Mann-Whitney test (4–6 repeats at each glucose concentration from 3 donors).

Once experimental conditions had been optimized we tested the bioenergetic response of our differentiated airway epithelial cells to altered glucose concentrations. We saw that following addition of both 5 mM and 15 mM d-glucose levels of ECAR significantly increased, when compared with 1 mM glucose while no changes were observed in OCRs in any of the glucose concentrations (Fig. 4*A*). Therefore, assuming mitochondrial CO_2_ production rates also remained the same, the changes in ECAR under these conditions would reflect the glycolysis dependent medium acidification rate. After the addition of oligomycin, the greatest decrease in OCR was seen in the 1 mM glucose samples.

Analysis of absolute ATP production rates showed that increasing the glucose concentration causes a significant and progressive increase in ATP production by glycolysis from 252 pmol/min at 1 mM to 703 pmol/min at 5 mM and 952 pmol/min at 15 mM (Fig. 4*B*). While glycolytic ATP production rates increased with increasing levels of glucose, we saw that mitochondrial (OXPHOS) ATP production rates significantly decreased from 798 pmol/min at 1 mM to 487 pmol/min at 5 mM and 362 pmol/min at 15 mM. There was no statistically significant difference in OXPHOS ATP production rates between the 5 mM and 15 mM glucose concentrations.

#### Proof-of-concept: inhibition of LDH suppresses glucose-induced extracellular acidification.

Multiple chronic lung diseases, including COPD, CF and IPF are associated with elevated lung lactate concentrations (26, 29), with the metabolic shifts toward glycolysis in the epithelium being at least in part implicated in these observed changes (7). To suppress lactate production by glycolysis in our fully differentiated ALI cultures we injected an inhibitor of LDH5 (Compound 408, Genentech) (Fig. 4*C*) (22). At all glucose concentrations there was significant decrease in the maximum ECAR after LDH5 inhibitor addition, with the greatest decrease at the highest concentration of 15 mM Glucose (Fig. 4*D*). Therefore, we have demonstrated empirically that LDH5 inhibition indeed lowered glycolytic activity of the cells.

## DISCUSSION

Our study presents a robust, practicable methodology that allows the metabolic characterization of differentiated primary human airway epithelial cell cultures using the widely available Seahorse XF24 Analyzer platform. This allows multiwell measurements of real time oxygen consumption and extracellular acidification rates in live cells, providing information on glycolysis and mitochondrial function. To our knowledge, this is the first time this has been possible using commercially available equipment.

This study used primary nasal epithelial cells as an exemplar for methodology development, due to their ability to replicate complex airway architecture in vitro (8). However, contrary to the previously proposed airways model (21), it is clear that there are differences between the upper and lower airway in their responses to disease relevant stimuli (4, 14) in addition to alterations in the cellular composition of the airway epithelium down the tracheobronchial tree. We suggest that our technique is broadly applicable to a range of airway cells grown on standard transwell support systems, and thus could be used to compare the metabolic profile of airway epithelial cells collected from different regions of the airway as well as comparisons between health and disease.

This method also has transferability across other disease models where cells are grown on transwell inserts, in particular where there is also a close association between altered glucose metabolism and disease risk (5, 15, 17). In principle this technique would open up additional avenues for metabolic research beyond epithelial monocultures, such as cocultures with fibroblasts which also undergo significant metabolic shifts in disease-relevant conditions (22). This would support development of novel therapeutic strategies for targeting epithelia-fibroblast crosstalk in idiopathic pulmonary fibrosis and related fibrotic interstitial lung diseases. Furthermore, because human variability is greater than that of animal models often used to study a disease, individual ex vivo metabolic profiling of the airway using our methods could provide vital information for personalized medicine (10). Such research could provide much needed translational insights and facilitate the development of novel therapeutic approaches.

We maintain that using ALI cultures is more physiologically relevant than using submerged cells, indicating the need for a method to transfer ALI cultures into the Seahorse Analyzer platform. The ALI cultures are highly differentiated, secretory, have motile cilia and therefore are more likely to recapitulate the complex metabolic requirements of airway epithelial cells in vivo. Basal epithelial cells, maintained in submerged culture, have a much lower rate of glycolysis and mitochondrial respiration than those grown at ALI (27) and therefore are not truly representative of in vivo airways. Furthermore, there are cell types which only become apparent when cells are maintained at ALI (20). Thus, using this novel methodology the Seahorse Analyzer is able to detect the integrated signal from the multiple cell types which are present in ALI cultures. This allows for the in vitro modeling of a physiologically heterogeneous whole airway epithelium rather than a single cell type.

We were able to show that in high, but physiologically relevant glucose conditions, ALI cultures significantly increased the glycolysis-dependent extracellular acidification rate. In vivo we believe that this could result in an acidification of the airway surface liquid (ASL). Recent research has suggested that the precise regulation of the ASL, in particular the pH, has a critical role in the prevention of infection and disease pathology (9, 11, 24). Therefore, the real time monitoring of pH and glucose metabolism, using platforms such as the Seahorse Analyzer, may be a key methodology for providing insights into the pathophysiology of airways disease and for therapeutic development. This may be especially relevant in situations where diabetes often represents an important comorbidity such as in cystic fibrosis, chronic obstructive pulmonary disease, and bronchiectasis.

As well as providing novel information relevant to the pathophysiology of airways disease, our study demonstrated proof of concept that cellular bioenergetics analysis of differentiated epithelia may provide insights in pharmaceutical development. A range of pathophysiological conditions including cancer and fibrotic lung diseases are thought to involve a reprograming of cellular metabolism, with skewing toward aerobic glycolysis, suggesting a potential therapeutic target. We showed that the addition of a potent LDH5 inhibitor resulted in a dose dependent reduction in extracellular acidification rate, consistent with a reduction in the production of lactate from pyruvate (22). Our system therefore allowed the evaluation of a novel compound in fully differentiated human cells, with the observed metabolic effects predicted from its designed mode of action.

In conclusion, we present a novel method and supporting data to demonstrate that it is possible to monitor the cellular metabolism in real time of fully ALI differentiated airway epithelial cells. The method also allowed us to monitor cellular responses to altered glucose concentrations, which may be relevant where diabetes and airway hyperglycemia can present as comorbidities to chronic airways diseases. We also found that it is possible to manipulate epithelial cell metabolism with a novel small molecule, indicating that this method may be useful for evaluating future therapeutic drug candidates.

## GRANTS

This work was funded by Medical Research Foundation (MRF Respiratory Diseases Research Award to J. P. Garnett; Grant MRF-091-0001-RGGARNE) and Boehringer Ingelheim. V. Saint-Criq was supported by CF Trust Strategic Research Centre grants (SRC003 and SRC013) and a Medical Research Council Confidence in Concept grant (MC_PC_15030).

## DISCLOSURES

No conflicts of interest, financial or otherwise, are declared by the authors.

## AUTHOR CONTRIBUTIONS

E.M., C.W., J.P.G., and S.M. conceived and designed research; E.M., B.V., and S.M. performed experiments; E.M., B.V., and S.M. analyzed data; E.M., J.P.G., and S.M. interpreted results of experiments; E.M. and B.V. prepared figures; E.M. drafted manuscript; E.M., B.V., S.C., V.S.-C., J.P., C.A.K., C.W., J.P.G., and S.M. edited and revised manuscript; E.M., B.V., S.C., V.S.-C., J.P., C.A.K., C.W., J.P.G., and S.M. approved final version of manuscript.
